# Therapeutic Effects of (5R)-5-Hydroxytriptolide on Fibroblast-Like Synoviocytes in Rheumatoid Arthritis *via* lncRNA WAKMAR2/miR-4478/E2F1/p53 Axis

**DOI:** 10.3389/fimmu.2021.605616

**Published:** 2021-02-16

**Authors:** Xinpeng Zhou, Duoli Xie, Jie Huang, Aiping Lu, Rongsheng Wang, Yehua Jin, Runrun Zhang, Cen Chang, Lingxia Xu, Linshuai Xu, Junyu Fan, Chao Liang, Dongyi He

**Affiliations:** ^1^Department of Rheumatology, Guanghua Hospital Affiliated to Shanghai University of Traditional Chinese Medicine, Shanghai University of Traditional Chinese Medicine, Shanghai, China; ^2^Department of Rheumatology, Shanghai Guanghua Hospital of Integrative Medicine, Shanghai, China; ^3^Department of Rheumatology, The Affiliated Hospital of Shandong University of Traditional Chinese Medicine (TCM), Jinan, China; ^4^School of Chinese Medicine, Law Sau Fai Institute for Advancing Translational Medicine in Bone and Joint Diseases, Hong Kong Baptist University, Hong Kong, China; ^5^Department of Biology, Southern University of Science and Technology, Shenzhen, China; ^6^Institute of Arthritis Research in Integrative Medicine, Shanghai Academy of Traditional Chinese Medicine, Shanghai, China; ^7^Guangdong-Hong Kong-Macau Joint Lab on Chinese Medicine and Immune Disease Research, Guangzhou University of Chinese Medicine, Guangzhou, China

**Keywords:** rheumatoid arthritis, (5R)-5-hydroxytriptolide, fibroblast-like synoviocytes, inflammation, WAKMAR2, miR-4478/E2F1/p53 axis

## Abstract

Rheumatoid arthritis (RA) is an autoimmune disease. Fibroblast-like synoviocytes (FLS) serve a major role in synovial hyperplasia and inflammation in RA. (5R)-5-hydroxytriptolide (LLDT-8), a novel triptolide derivative, shows promising therapeutic effects for RA and is now in phase II clinical trials in China. However, the underlying mechanism of LLDT-8 is still not fully understood. Here, we found that LLDT-8 inhibited proliferation and invasion of RA FLS, as well as the production of cytokines. Microarray data demonstrated that LLDT-8 upregulated the expression of long non-coding RNA (lncRNA) WAKMAR2, which was negatively associated with proliferation and invasion of RA FLS, as well as the production of pro-inflammatory cytokines. Knockdown of WAKMAR2 abolished the inhibitory effects of LLDT-8 on RA FLS. Mechanistically, WAKMAR2 sponged miR-4478, which targeted E2F1 and downstreamed p53 signaling. Rescue experiments indicated that the inhibitory effects of LLDT-8 on RA FLS were dependent on WAKMAR2/miR-4478/E2F1/p53 axis.

## Introduction

Rheumatoid arthritis (RA) is a common chronic autoimmune disease characterized by persistent synovitis, systemic inflammation, and joint destruction (1, 2). The synovium is transformed into a hyperplastic tissue in patients with RA (3). Fibroblast-like synoviocytes (FLS) are the most common cells at the pannus-cartilage junction and serve a major role in the hyperplastic process of synovium (4). FLS exhibit unique features with aggressive and invasive properties in RA, which are tumor-like phenotypes (5). FLS invade joint cartilage and contribute to joint destruction through the production of pro-inflammatory cytokines, chemokines, and matrix-degrading molecules (6). Targeting FLS is emerging as an attractive therapeutic approach in the RA treatment (3).

Triptolide, a structurally unique diterpenoid obtained from *Tripterygium wilfordii* Hook F, has excellent efficiency in fighting against cancers and RA (7). However, low aqueous solubility, tissue accumulation, and toxicity limit the clinical use of triptolide (8). (5R)-5-Hydroxytriptolide (LLDT-8) is a novel triptolide derivative (9). Compared to triptolide, LLDT-8 has a better safety profile and does not induce abnormalities in epididymis, liver, kidney, spleen, and circulation (10). Furthermore, LLDT-8 has comparable immunosuppressive activity to Triptolide. Thus, LLDT-8 is suggested to be an optimal analog of triptolide (11). Studies have reported that LLDT-8 prevents collagen-induced arthritis (CIA) in animal models (12, 13). LLDT-8 is now in phase II clinical trials, in China, for RA treatment (14). However, the underlying mechanism of LLDT-8 is still not fully understood.

Accumulating evidence has suggested that long non-coding RNAs (lncRNAs), which modulate the gene expression through multiple mechanisms, are important molecules involved in immune and inflammatory pathways in RA (15, 16). This preliminary study showed that LLDT-8 induced substantial changes of lncRNAs in RA FLS (17). In this study, we examined the effects of LLDT-8 on proliferation and invasion of RA FLS, as well as the production of pro-inflammatory cytokines. We determined LLDT-8-induced changes of lncRNAs and identified that WAKMAR2 (also named as ENST00000606327) mediated the effects of LLDT-8 on RA FLS. We predicted downstream miRNAs and signaling pathways and validated that LLDT-8 acted through WAKMAR2/miR-4478/E2F1/p53 axis in RA FLS.

This study revealed that LLDT-8 exerted inhibitory effects on proliferation and invasion of RA FLS, as well as the production of cytokines *via* WAKMAR2/miR-4478/E2F1/p53 axis.

## Materials and Methods

### Isolation of RA FLS

Synovial tissues were obtained from patients with RA who underwent total knee joint replacement at Guanghua Hospital (Shanghai, China). All the patients fulfilled the American College of Rheumatology 1987 revised classification criteria for RA (18). The study was approved by the Ethical Committee of Guanghua Hospital, and all participants signed informed consent prior to participation. RA FLS were isolated from synovial tissues as described previously (17). Briefly, joint tissues were minced into pieces and treated with 2–4 mg/ml of collagenase (Serva) for 2 h in DMEM at 37°C. Dissociated cells were then centrifuged and re-suspended in Dulbecco's Modified Eagle's medium (DMEM) (Gibco) supplemented with 10% FBS and 1% penicillin-streptomycin. The cells were kept at 37°C in 5% CO_2_, and the culture medium was replaced every 2–3 days. RA FLS at passages 3–5 were used in the experiments. No mycoplasma contamination was detected. The purity of the cells was verified by fluorescence activated cell sorting (FACS) using specific cell-surface markers, including CD68 (a marker of macrophages) and CD90 (a marker of fibroblasts) (16, 19). The cells were activated by incubating with 10 ng/ml TNF-α and 10 ng/ml IL-17 (Proteintech) for 12 h before treating with LLDT-8 (50 or 100 nm, Shanghai Pharma), vehicle (DMSO), miRNA mimics, antagomir, lncRNA knockdown, or overexpression vectors (20, 21).

### Characterization of RA FLS Using FACS

Briefly, 1 × 10^6^ cells were counterstained with 1 μg/ml of propidium iodide (Thermo Fisher Scientific), and non-viable cells were excluded from living cells. Monoclonal antibodies, including APC-labeled CD90 and PE-labeled CD68 (BD Biosciences), were used for the characterization of FLS. Each experiment contained isotype-matched control antibodies. Flow cytometric analysis was performed on a BD FACSAria™ III Cell Sorter (BD Biosciences).

### Isolation of T Cells and Monocytes/Macrophages and Cell Co-culture

Peripheral blood mononuclear cells (PBMCs) were obtained from freshly drawn anticoagulated whole blood of healthy volunteers using a MACSprep™ PBMC Isolation kit (Miltenyi Biotec, Bergisch Gladbach, Germany). The CD4^+^ T cells were enriched with PBMCs by magnetic bead sorting (Miltenyi Biotec), labeled with CellTrace^TM^ Violet (CTV, Thermo Fisher Scientific) and co-cultured with RA FLS at a 2:1 ratio in the presence of anti-CD3 and anti-CD28 stimulation for 3 days. The proliferation of CTV-labeled CD4^+^ T cells was examined by flow cytometry (22). The CD14^+^monocytes/macrophages were enriched with PBMCs by magnetic bead sorting (Miltenyi Biotec) and co-cultured with RA FLS at a 2:1 ratio for 3 days. Osteoclastic differentiation of CD14^+^monocytes/macrophages was evaluated by examining the tartrate resistant acid phosphatase (TRAP) level using ELISA (23).

### Construction of Plasmids and Production of Lentivirus

The pHBLV-U6-MCS-CMV-ZsGreen-PGK-Puro is the lentiviral vector that was used for silencing WAKMAR2. The other lentiviral vector that was used for the overexpression of WAKMAR2 was pHBLV-CMV-MCS-EF1-Zsgreen1-T2A-Puro. The above vectors and packaging plasmids, such as psPAX2 and pMD2G, were co-transfected into 293T cells using a Lipofiter^TM^ transfection reagent (Hanbio). The medium containing lentivirus was collected at 48 h and at 72 h. The lentiviral particles were concentrated, as previously described, and stored in cryovials at −80°C until use (24, 25).

### Real-Time PCR

Relative expression levels of lncRNAs and miRNAs in RA FLS were determined by real-time PCR (26). Total RNA was isolated by a TRIzol reagent and quantified with NanoDrop ND-2000 (Thermo Scientific). Quality control was performed by Agilent Bioanalyzer 2100 (Agilent Technologies Inc.). The RNA was reverse transcribed by a PrimeScript^TM^ RT reagent kit containing a gDNA eraser (Takara). Real-time PCR was conducted with a SYBR Premix Ex TaqTM kit (TliRNaseH Plus) in an Mx3005P qPCR System (Agilent Technologies Inc.). Glyceraldehyde 3-phosphate dehydrogenase (GAPDH) was used as an internal control. The primers used were listed below: WAKMAR2: 5′-GGCCTCAGTGAGGTAAATCG-3′; 5′-CATACCACTACACTCCAGC-3′ and GAPDH: 5′-AACTTTGGCATTGTGGAAGG-3′; 5′-GGATGCAGGGATGATGTTCT-3′. The expressions of miRNAs were detected by stem-loop real-time PCR. The miRNAs from total RNA were reverse transcribed and subjected to amplification using Hairpin-it™ MicroRNAs Quantitation kits (GenePharma), which contained both primers of miRNAs and the internal control U6. The obtained data were analyzed by the MXProv 4.1 Sequence Detection System and calculated according to the 2^−Δ*ΔCt*^ formula (27).

### Cell Counting Kit-8 (CCK-8) Assay

Fibroblast-like synoviocytes were seeded in 96-well-plates at a density of 2 × 10^3^ cells per well. The cell proliferation index was measured using a CCK-8 assay (Dojindo) at 0, 24, 48, 72, and 96 h after the cells were seeded. A volume of 10 μl CCK-8 reagent and 90 μl DMEM (Gibco) were added to every well and incubated at 37°C for 1.5 h. Cell proliferation was evaluated at 450 nm by reading the optical density (OD) value using a SpectraMax 190 Microplate Reader (Molecular Devices).

### Colony Formation Assay

Exponentially growing RA FLS were collected and seeded in 12-well-plates at a density of 2 × 10^2^ cells per well. The medium was changed every 2–3 days for 1–2 weeks and was terminated when macroscopic apophyses were found. The colonies were stained with 0.2% crystal violet for 30 min after fixation with 4% paraformaldehyde for 15 min. The number of colonies was scored on a cloning counter (28).

### Transwell Assay

RA FLS were harvested and suspended in DMEM medium without FBS. In the upper chamber, 1 × 10^3^ cells/well was seeded in a medium, without FBS, containing a polycarbonate membrane coated with Matrigel. Another medium containing 10% FBS was added to the lower chamber. The non-invading cells in the upper chamber were removed by cotton swabs after incubation at 37°C for 48 h. Cells that had passed through the membrane were stained with crystal violet after being fixed with 4% paraformaldehyde. Photographs were captured in three different fields, and the number of cells that invaded through the membrane was counted (29).

### ELISA

Cell supernatant was collected, and the amount of interleukin-1 (IL-1), interleukin-6 (IL-6), tartrate-resistant acid phosphatase (TRAP), and matrix metalloproteinases-3 (MMP-3) was quantitated using ELISA kits for IL-1 (Immunoway), IL-6 (Immunoway), TRAP (Invitrogen), and MMP-3 (Immunoway) in accordance with the manufacturer's instructions, respectively. ELISA plates were analyzed with a SpectraMax 190 Microplate Reader (Molecular Devices) (30).

### Immunoblotting

Total protein was extracted from RA FLS. A BCA kit (Beyotime) was used for evaluating the total protein concentration. Protein samples were separated by sodium dodecyl sulfate-polyacrylamide gel electrophoresis (SDS-PAGE) and transferred onto PVDF membranes (Millipore). After blocking, the membranes were probed with primary antibodies and then incubated with specific horseradish peroxidase-conjugated secondary antibodies (Bio-Rad). Immunodetection was performed using an enhanced chemiluminescence kit (Thermo Fisher Scientific). GAPDH was used as a loading control for internal correction. The primary antibodies included an anti-proliferating cell nuclear antigen (PCNA) antibody (Abcam), an anti-cyclin D1 antibody (Abcam), an anti-IL-1 antibody (Abcam), an anti-IL-6 antibody (Abcam), an anti-MMP-3 antibody (Abcam), an anti-p53 antibody (Abcam), an anti-E2F1 antibody (Abcam), anti-ErbB1/2 antibodies (Abcam), and a GAPDH antibody (Sigma). The bands were exposed under x-ray using Gel Imager (Bio-Rad) (17).

### Dual-Luciferase Reporter Assay

The pmirGLO Dual-Luciferase vectors were used (Promega) for luciferase reporter assay. The wild-type E2F1 3′UTR or WAKMAR2 containing miR-4478 binding sequences were inserted into the pmirGLO vectors to produce WT-WAKMAR2 and WT-E2F1, respectively. The Mut-WAKMAR2 and Mut-E2F1 with mutated binding sites were also generated using the same method. The miR-4478 mimic or NC mimic were co-transfected with any of the following, such as WT-WAKMAR2, Mut-WAKMAR2, WT-E2F1, and Mut-E2F1, into cells using Lipofectamine 3000 (Invitrogen). Luciferase reporter assays were performed according to the manufacturer's instructions (Promega). Firefly and Renilla luciferase activities were examined by the Dual-Luciferase Reporter Assay System, and the firefly activity was normalized to Renilla activity.

### Fluorescence *in situ* Hybridization (FISH) Assay

Subcellular localization of WAKMAR2 in RA FLS was examined using a FISH assay (31, 32). Fluorescence probe was designed by Guge Biotechnology Co. Ltd. The probe sequence was 5′-FAM-TTCTTCATGAGGAGAAACTCAATGAGGAAATTTGTG-3′. RA FLS were seeded in 20 mm nest (1 × 10^5^ cells/nest) and fixed in 4% paraformaldehyde for 15 min. Cells were washed and permeabilized for 15 min at 4°C. Then, the cells were dehydrated using 75, 85, and 100% ethanol for 1 min, respectively, followed by incubation with probe in hybridization buffer overnight in 37°C. The non-specific binding of probes was blocked through incubation in 3% bovine serum albumin (BSA). The cells were counterstained with DAPI for 10 min and visualized by confocal microscopy (Leica Microsystems).

### The miRNA Microarray

Expression profiles of miRNAs were accessed using a microarray. Briefly, total RNA was extracted with a TRIzol reagent; the concentration and integrity were assessed by Nanodrop ND-2000 (Thermo Fisher Scientific) and Bioanalyzer 2100 (Agilent Technologies). RNA was transcribed to double-stranded cDNA, synthesized into cRNA, and labeled with Cyanine-3-CTP. The labeled cRNAs were then hybridized onto the microarray. After washing, the arrays were scanned by the Agilent Scanner G2505C (Agilent Technologies) and analyzed by Feature Extraction Software (version 10.7.1.1, Agilent Technologies). Other processing procedures like sample labeling, hybridization, fluidics processing, and scanning were also performed according to the manufacturer's standard protocols (33). Differentially expressed miRNAs were identified by a fold change ≥2.0 and *P* ≤ 0.05. Gene ontology (GO) analysis was performed to identify biological functions, which covers the biological process, the cellular component, and the molecular function. The Kyoto Encyclopedia of Genes and Genomes (KEGG) pathway analysis was applied to determine the roles of the differentially expressed mRNAs in biological pathways.

### Statistical Analysis

All statistical analyses were conducted using GraphPad 6.02 Software. Comparison between two groups was detected using Student's *t*-test, while for difference among multiple independent groups, a one-way analysis of variance (ANOVA) with a *post-hoc* test was performed. The data were presented as mean ± SD and *P* < 0.05 was considered significant.

## Results

### Therapeutic Effects of LLDT-8 on RA FLS

**Figure 1:**
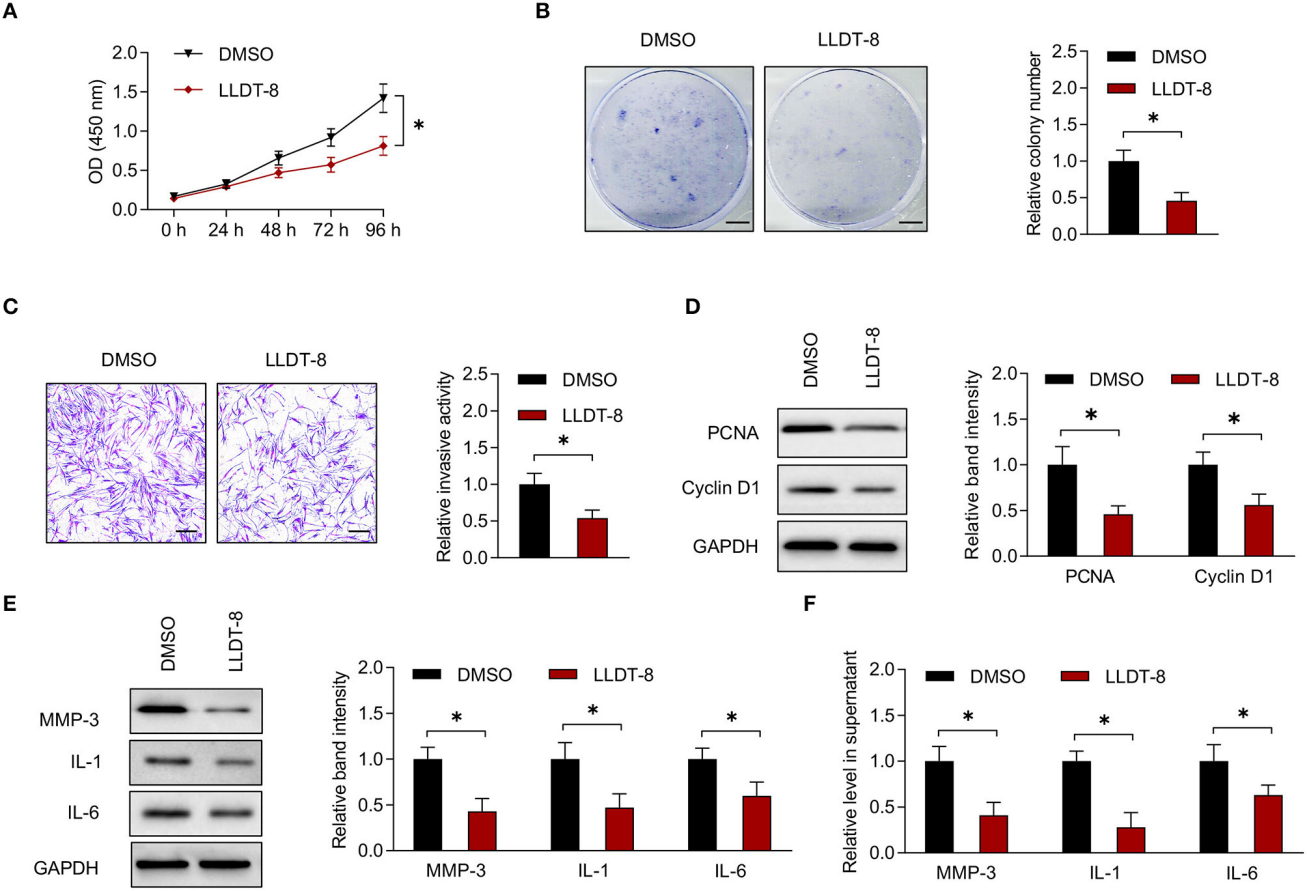
Effects of LLDT-8 on RA FLS. **(A)** Proliferative ability of RA FLS treated with 50 nm LLDT-8 or vehicle (DMSO), as determined by CCK-8 assay. **(B)** Colony formation of RA FLS with the above treatment. Scale bars = 5 mm. **(C)** Invasion of RA FLS with the above treatment, as determined by transwell assay. Scale bars = 200 μm. **(D)** The levels of PCNA and Cyclin D1 in RA FLS with the above treatment, as determined by western blotting. **(E)** The levels of MMP-3, IL-1, and IL-6 in RA FLS with the above treatment, as determined by western blotting. **(F)** The levels of MMP-3, IL-1, and IL-6 in supernatant of RA FLS with the above treatment, as determined by ELISA. The data are presented as mean ± SD. *P < 0.05. Both, representative images and quantitative measurement of colony formation, invasion, and western blotting were shown. All experiments were repeated 3 times. LLDT-8, (5R)-5-hydroxytriptolide; RA, rheumatoid arthritis; FLS, fibroblast-like synoviocytes; DMSO, dimethyl sulfoxide; PCNA, proliferating cell nuclear antigen; ELISA, enzyme-linked immunosorbent assay; IL, interleukin; MMP, matrix metalloproteinase.

Fibroblast-like synoviocytes were isolated from patients with RA and characterized by cell surface markers including the positive expression of CD90 and the absence of macrophage marker CD68 (16, 19). Flow cytometric analysis showed that the expression rate of CD90 was 93% when compared to isotype control. On the contrary, the expression of CD68 was negative (Supplementary Figure 1A). We incubated RA FLS with LLDT-8 or vehicle (DMSO) *in vitro*. The CCK-8 and colony formation assay showed that LLDT-8 inhibited proliferation of RA FLS when compared to DMSO (Figures 1A,B). Transwell assay demonstrated that LLDT-8 decreased the invasion of RA FLS (Figure 1C). Cell cycle proteins including PCNA and Cyclin D1 are direct readouts of cellular proliferation status (34, 35). FLS incubated with LLDT-8 showed lower expression of PCNA and Cyclin D1 (Figure 1D). The MMPs and pro-inflammatory cytokines produced by RA FLS have pivotal roles in the destruction of cartilage and bone in RA (36). After incubation with LLDT-8, there were lower expression and release of MMP-3 and pro-inflammatory cytokines (IL-1 and IL-6) in RA FLS (Figures 1E,F). RA FLS also aid in the activation of immune responses by interacting with immune cells, such as T cells and macrophages. The RA FLS stimulate CD4^+^ T cell proliferation *via* cytokine production (22, 37). They interact with macrophages to induce osteoclast differentiation (37). The RA FLS were treated with LLDT-8 or DMSO and co-cultured with CD4^+^ T cells and CD14^+^ monocytes/macrophages, respectively. Proliferation of CD4^+^ T cells and expression of TRAP (a marker of osteoclast differentiation) (23) in monocytes/macrophages were less significant after being co-cultured with LLDT-8-treated RA FLS when compared to those after being co-cultured with DMSO-treated RA FLS (Supplementary Figure 1B).

### Upregulation of WAKMAR2 Induced by LLDT-8

**Figure 2:**
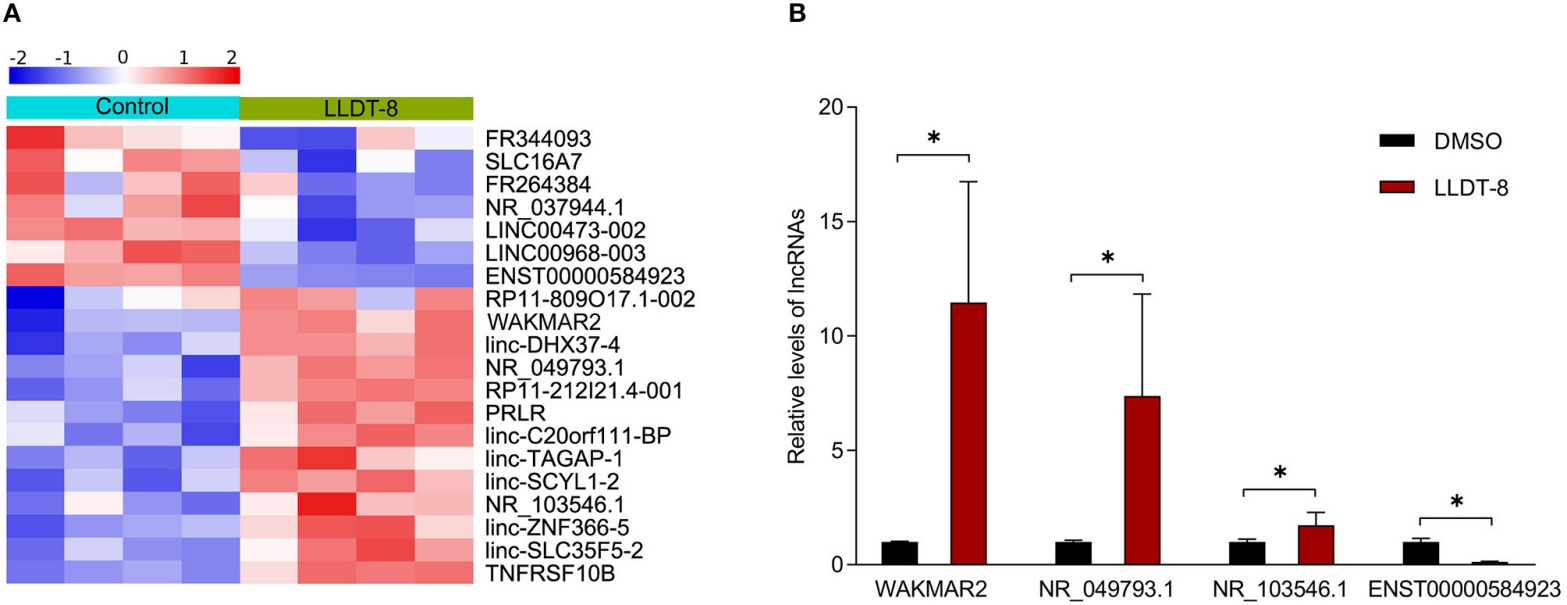
Effects of LLDT-8 on expression of lncRNAs in RA FLS. **(A)** Heatmap showing differentially expressed lncRNAs in RA FLS treated with LLDT-8 or DMSO. **(B)** Relative levels of WAKMAR2, NR_049793.1, NR_103546.1, and ENST00000584923 in RA FLS treated with LLDT-8 or DMSO, as determined by real-time PCR. The data are presented as mean ± SD. *P < 0.05. All experiments were repeated 8 times. LLDT-8, (5R)-5-hydroxytriptolide; RA, rheumatoid arthritis; FLS, fibroblast-like synoviocytes; lncRNAs, long non-coding ribonucleic acids; DMSO, dimethyl sulfoxide; PCR, polymerase chain reaction.

In a previous study, we found that LLDT-8 induced substantial changes of lncRNAs in RA FLS (17). We chose differentially expressed lncRNAs for experimental validation (Figure 2A). After treatment with LLDT-8 or DMSO, the expression of lncRNAs in RA FLS was determined by real-time PCR. Our results showed that WAKMAR2, NR_049793.1, and NR_103546.1 were upregulated in RA FLS treated with LLDT-8, while ENST00000584923 was downregulated, when compared to cells treated with DMSO. We chose WAKMAR2 for further investigation, as the upregulation of WAKMAR2 induced by LLDT-8 was the most significant (Figure 2B).

### Role of WAKMAR2 in RA FLS

**Figure 3:**
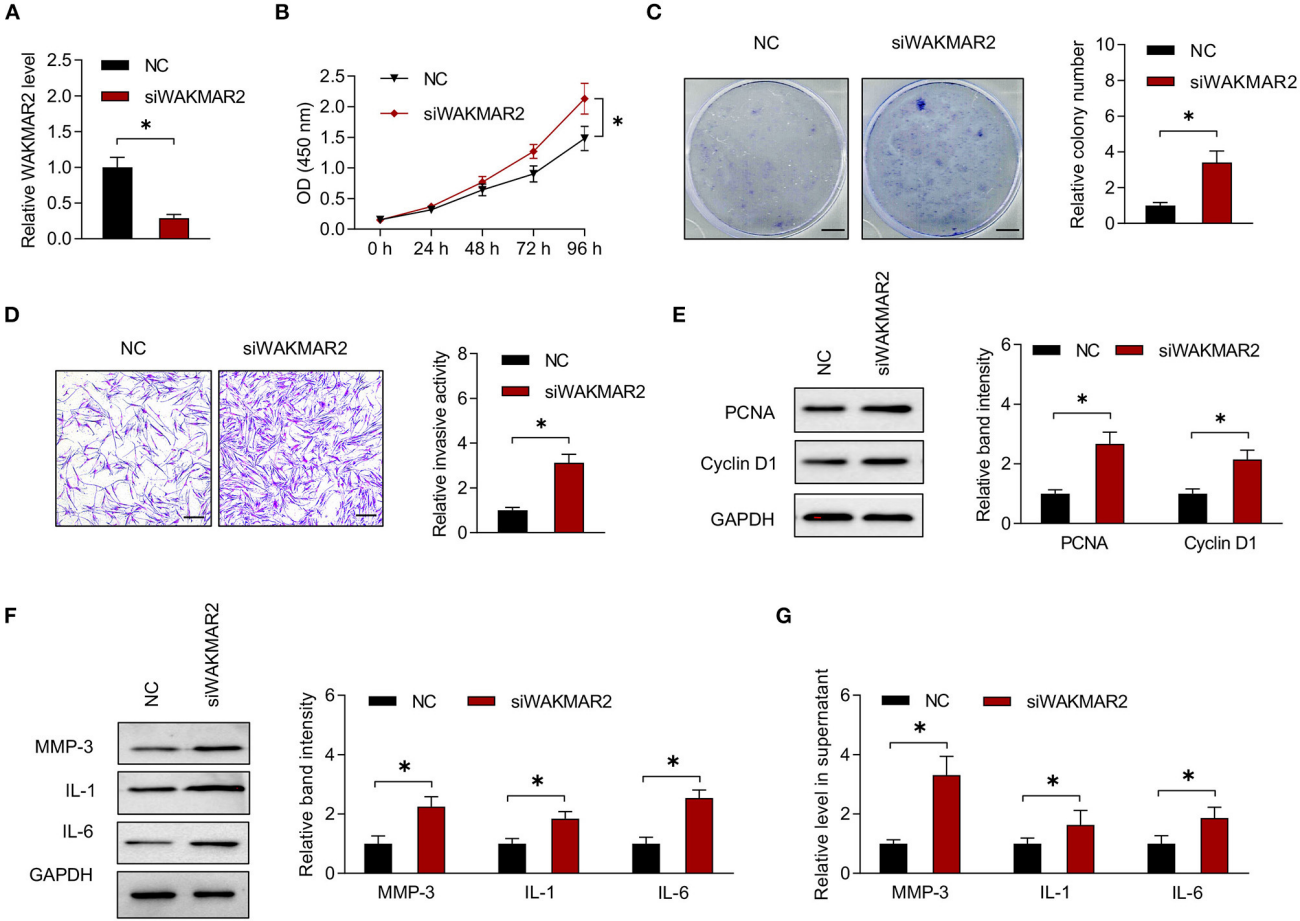
Effects of WAKMAR2 silencing on RA FLS. **(A)** Levels of WAKMAR2 in RA FLS transfected with WAKMAR2 silencing vector (siWAKMAR2) or negative control vector (NC), as determined by real-time PCR. **(B)** Proliferative ability of RA FLS with the above transfection, as determined by CCK-8 assay. **(C)** Colony formation of RA FLS with the above transfection. Scale bars = 5 mm. **(D)** Invasion of RA FLS with the above transfection, as determined by transwell assay. Scale bars = 200 μm. **(E)** The levels of PCNA and Cyclin D1 in RA FLS with the above transfection, as determined by western blotting. **(F)** The levels of MMP-3, IL-1, and IL-6 in RA FLS with the above transfection, as determined by western blotting. **(G)** The levels of MMP-3, IL-1, and IL-6 in supernatant of RA FLS with the above transfection, as determined by ELISA. The data are presented as mean ± SD. *P < 0.05. Both, representative images and quantitative measurement of colony formation, invasion and western blotting were shown. All experiments were repeated 3 times. RA, rheumatoid arthritis; FLS, fibroblast-like synoviocytes; NC, negative control vector; PCR, polymerase chain reaction; PCNA, proliferating cell nuclear antigen; IL, interleukin; MMP, matrix metalloproteinase; ELISA, enzyme-linked immunosorbent assay.

To determine the role of WAKMAR2 in RA FLS, we performed gene silencing or overexpression of WAKMAR2 (Figure 3A and Supplementary Figure 2A). Then, we determined the proliferation and invasion of RA FLS, as well as the production of MMP-3, IL-1, and IL-6. The CCK-8 and colony formation assay showed that the silencing of WAKMAR2 promoted proliferation of RA FLS (Figures 3B,C). Transwell assay demonstrated that silencing of WAKMAR2 enhanced the invasion of RA FLS (Figure 3D). Silencing of WAKMAR2 increased the levels of PCNA and Cyclin D1 and enhanced the expression and secretion of MMP-3, IL-1, and IL-6 (Figures 3E–G). In contrast, WAKMAR2 overexpression inhibited the proliferation and invasion of RA FLS (Supplementary Figures 2B–D), reduced the levels of PCNA and Cyclin D1 (Supplementary Figure 2E), and decreased the expression and secretion of MMP-3, IL-1, and IL-6 (Supplementary Figures 2F,G). The RA FLS with WAKMAR2 silencing or overexpression were co-cultured with CD4^+^ T cells and CD14^+^ monocytes/macrophages, respectively. Proliferation of CD4^+^ T cells and expression of TRAP in monocytes/macrophages were higher after they were co-cultured with RA FLS with WAKMAR2 silencing (Supplementary Figure 3A), whereas the proliferation of CD4^+^ T cells and the expression of TRAP in monocytes/macrophages were lower after they were co-cultured with RA FLS with WAKMAR2 overexpression (Supplementary Figure 3B).

### Effects of LLDT-8 on RA FLS With WAKMAR2 Silencing

**Figure 4:**
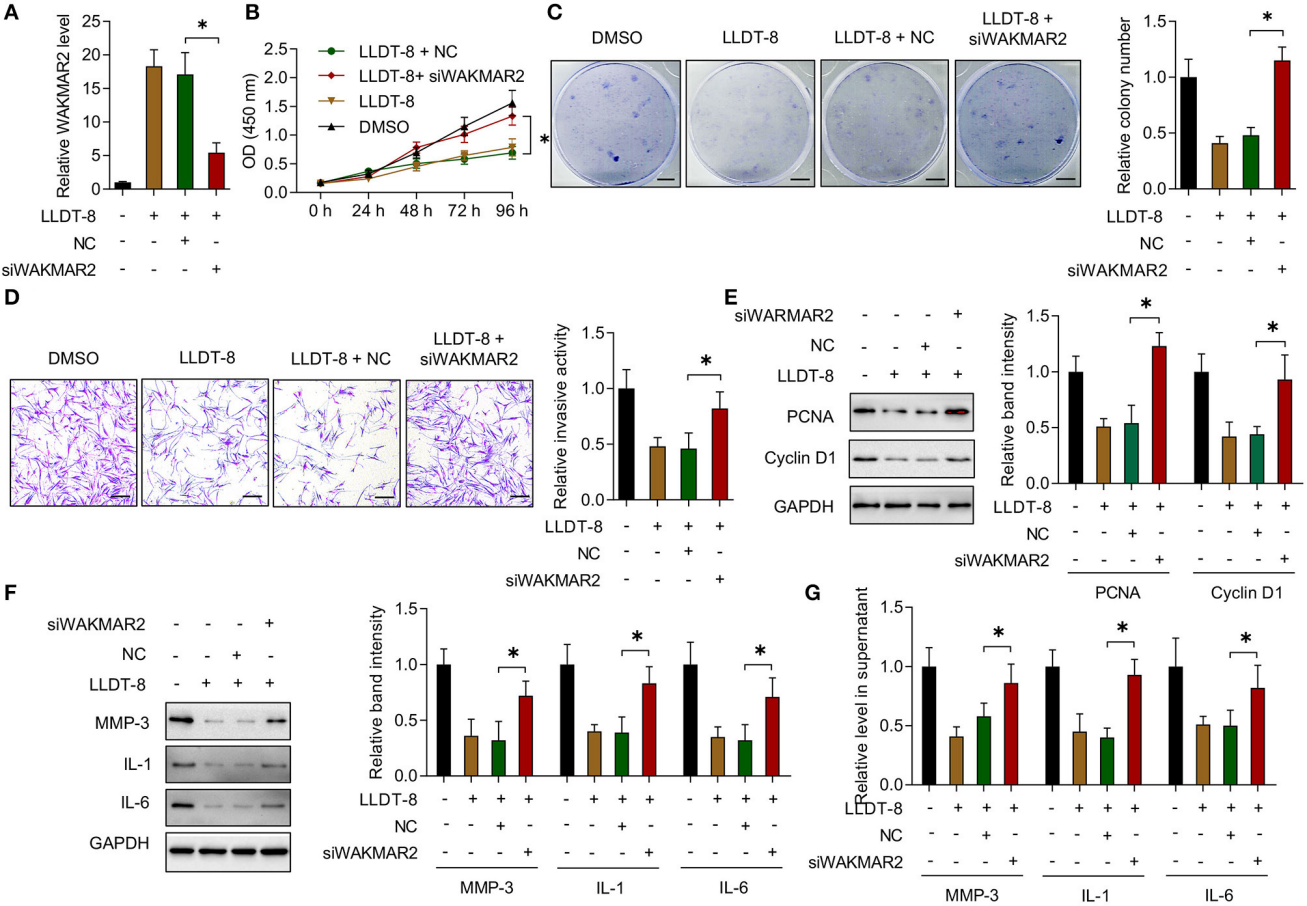
Effects of LLDT-8 on RA FLS with WAKMAR2 silencing. **(A)** Level of WAKMAR2 in LLDT-8-treated FLS with or without transfection of WAKMAR2 silencing vector (siWAKMAR2) or negative control vector (NC), as determined by real-time PCR. **(B)** Proliferative ability of RA FLS with the above treatment, as determined by CCK-8 assay. **(C)** Colony formation of RA FLS with the above treatment. Scale bars = 5 mm. **(D)** Invasion of RA FLS with the above treatment, as determined by transwell assay. Scale bars = 200 μm. **(E)** The levels of PCNA and Cyclin D1 in RA FLS with the above treatment, as determined by western blotting. **(F)** The levels of MMP-3, IL-1, and IL-6 in RA FLS with the above treatment, as determined by western blotting. **(G)** The levels of MMP-3, IL-1, and IL-6 in supernatant of RA FLS with the above treatment, as determined by ELISA. The data are presented as mean ± SD. *P < 0.05.Both, representative images and quantitative measurement of colony formation, invasion, and western blotting were shown. All experiments were repeated 3 times. RA, Rheumatoid arthritis; FLS, Fibroblast-like synoviocytes; PCNA, proliferating cell nuclear antigen; IL, interleukin; MMP, matrix metalloproteinase; ELISA, enzyme-linked immunosorbent assay.

The RA FLS with or without WAKMAR2 silencing were treated with LLDT-8 *in vitro* (Figure 4A). In the presence of WAKMAR2 silencing, LLDT-8 could not inhibit the proliferation of RA FLS, when compared to cells without WAKMAR2 silencing (Figures 4B,C). Invasion of RA FLS with both WAKMAR2 silencing and LLDT-8 treatment was remarkable when compared to cells treated with LLDT-8 alone (Figure 4D). There was a higher expression of PCNA and Cyclin D1 in RA FLS with both WAKMAR2 silencing and LLDT-8 treatment (Figure 4E). We also detected that WAKMAR2 silencing alleviated LLDT-8-induced inhibition of MMP-3, IL-1, and IL-6 expression and secretion in RA FLS (Figures 4F,G).

### The GO and KEGG Pathway Enrichment Analyses

**Figure 5:**
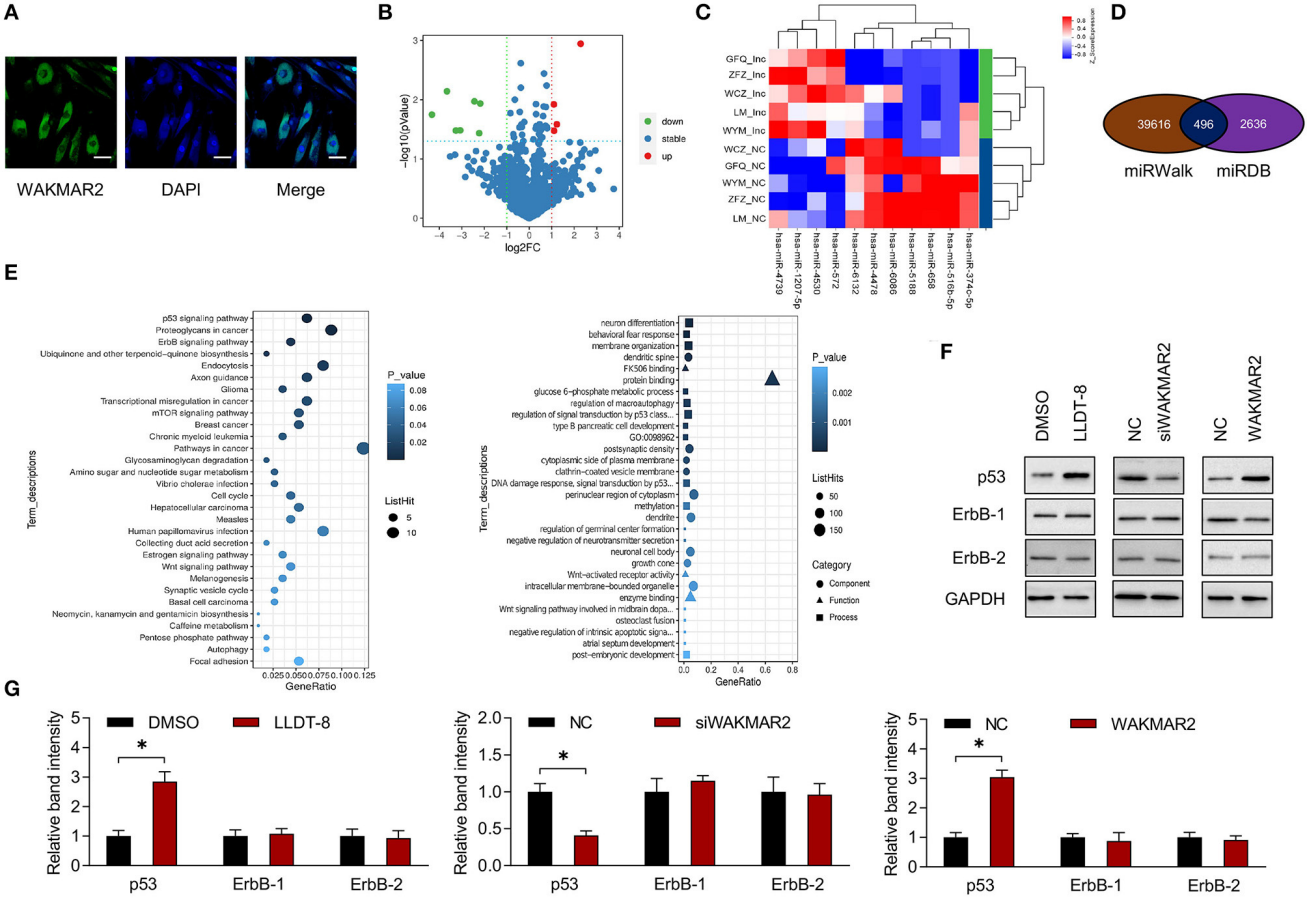
Expression profiling of miRNAs and bioinformatic analysis. **(A)** Subcellular localization of WAKMAR2 in RA FLS treated with 100 nM LLDT-8, as determined by FISH assay. Green color showed localization of WAKMAR2. Blue color showed nuclear staining by DAPI. Scale bars= 50 μm. **(B)** Volcano plot showing differentially expressed miRNAs in RA FLS with overexpression of WAKMAR2. Red color indicated upregulated miRNAs and green color indicated downregulated miRNAs after defining the threshold of fold change ≥2 and *P* < 0.05. **(C)** Heatmap illustrating expression patterns of the significantly upregulated and downregulated miRNAs. **(D)** Overlapping miRNA-mRNA pairs between miRWalk and miRDB databases. **(E)** GO and KEGG pathway enrichment analysis. The top 30 items with the smallest *P*-value in the enrichment analysis were shown in KEGG (left) and GO (right) bubble diagrams. **(F)** The levels of p53 and ErbB-1/2 in RA FLS treated with LLDT-8 or DMSO, or transfected with WAKMAR2 silencing vector (siWAKMAR2), WAKMAR2 overexpressing vector or their corresponding negative control vectors (NC). **(G)** Quantitative measurement of western blotting. The data are presented as mean ± SD. *P < 0.05. All experiments were repeated 3 times. FISH, fluorescence *in situ* hybridization; miRNA, microRNA; RA, rheumatoid arthritis; FLS, fibroblast-like synoviocytes; GO, gene ontology; KEGG, kyoto encyclopedia of genes and genomes.

To examine the subcellular localization of WAKMAR2, the RA FLS were treated with LLDT-8. The FISH assay showed that WAKMAR2 were mainly present in the cytoplasm (Figure 5A). We performed miRNA microarray in RA FLS with overexpression of WAKMAR2 and 539 miRNAs were detected (Figure 5B). We analyzed the statistical significance of differentially expressed miRNAs with the threshold of fold change ≥2 and *P* < 0.05, and obtained 11 miRNA candidates, including four upregulated miRNAs and seven downregulated miRNAs, as shown in a volcano plot and a heatmap (Figures 5B,C). We predicted target mRNAs of miRNAs using miRWalk and miRDB databases. 496 overlapping miRNA-mRNA pairs between the two databases were identified (Figure 5D). We performed GO and KEGG pathway enrichment analyses of the mRNAs. The most enriched pathways included p53 and ErbB (Figure 5E), which were related to cellular proliferation, invasion of RA FLS, and production of cytokines (38–40). We examined the effects of LLDT-8 treatment, WAKMAR2 silencing, and overexpression of p53 and ErbB levels in RA FLS, respectively. The LLDT-8 treatment induced an increased expression of p53 rather than ErbB-1 and ErbB-2. The WAKMAR2 overexpression mimicked the effects of LLDT-8 treatment, while WAKMAR2 silencing decreased the expression of p53 rather than ErbB-1 and ErbB-2 (Figures 5F,G).

### Interaction Between WAKMAR2 and miR-4478

**Figure 6:**
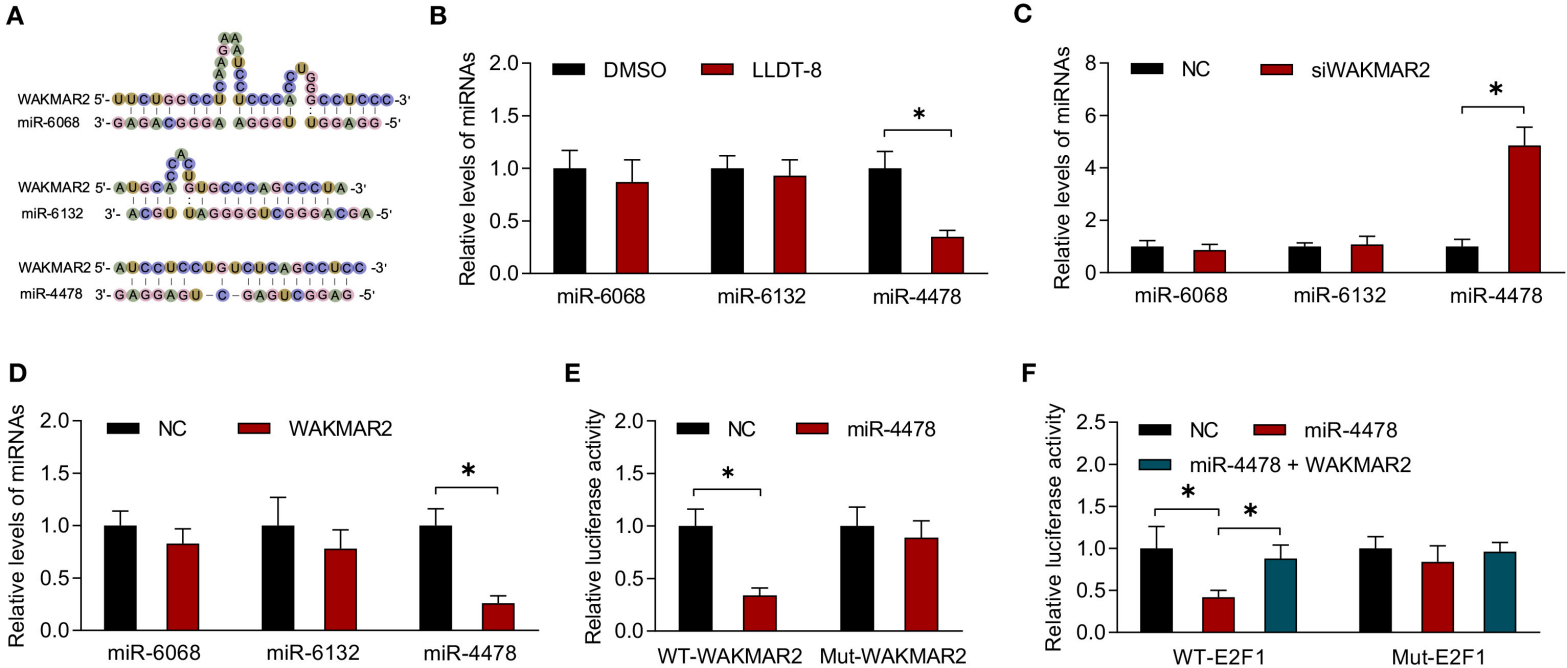
Interaction between miR-4478 and WAKMAR2. **(A)** Prediction of binding sites between WAKMAR2 and miRNA candidates (miR-6068, miR-6132, and miR-4478) using RNAHybrid. **(B)** Relative levels of miR-6068, miR-6132, and miR-4478 in RA FLS treated with LLDT-8 or DMSO. **(C)** Relative levels of miR-6068, miR-6132, and miR-4478 in RA FLS transfected with WAKMAR2 silencing vector (siWAKMAR2) or negative control vectors (NC). **(D)** Relative levels of miR-6068, miR-6132, and miR-4478 in RA FLS transfected with WAKMAR2 overexpressing vector or negative control vectors (NC). **(E)** Luciferase activity in RA FLS transfected with miR-4478 mimic or negative control mimic (NC) and WT-WAKMAR2 or Mut-WAKMAR2, as determined by luciferase reporter assay. **(F)** The luciferase activity in RA FLS transfected with miR-4478 mimic, negative control mimic (NC), or miR-4478 mimic + WAKMAR2 overexpression vector and WT-E2F1 or Mut-E2F1. Data are presented as mean ± SD. *P < 0.05. All experiments were repeated 3 times. RNA, ribonucleic acid; RA, rheumatoid arthritis; FLS, fibroblast-like synoviocytes; NC, negative control vector.

The lncRNAs could sponge miRNAs or function as competing endogenous RNAs (ceRNAs) by occupying the shared binding sequences of miRNAs, thus sequestering miRNAs and changing the expression of downstream target genes (41). We analyzed the binding sites between WAKMAR2 and the seven downregulated miRNAs using RNAhybrid. The three miRNAs (miR-4478, miR-6068, and miR-6132) containing potential binding sites with WAKMAR2 were chosen for experimental validation (Figure 6A).We examined the effects of LLDT-8 on the expression of miR-4478, miR-6068, and miR-6132 in RA FLS. LLDT-8 dramatically decreased the miR-4478 level and did not affect the expression of miR-6068 and miR-6132 (Figure 6B). We also conducted gene silencing of WAKMAR2 in RA FLS and found an increased level of miR-4478 and unaltered expression of miR-6068 and miR-6132 (Figure 6C). Overexpression of WAKMAR2 reduced the level of miR-4478 and it had no effect on the expression of miR-6068 and miR-6132 (Figure 6D). To confirm the targeting relationship between WAKMAR2 and miR-4478, luciferase reporter assay was performed based on the predicted binding site. The results showed that the overexpression of miR-4478 significantly inhibited the luciferase activity of WT-WAKMAR2 rather than Mut-WAKMAR2 (Figure 6E). It has been reported that miR-4478 could target E2F1 (42). The E2F1 activates p53 transcription and regulates the cell cycle (43, 44). We determined whether E2F1 was involved in WAKMAR2-mediated ceRNA network. The luciferase reporter assay illustrated that the overexpression of miR-4478 reduced the luciferase activity of WT-E2F1 rather than Mut-E2F1 (Figure 6F). The enforced expression of WAKMAR2 reversed the reduction of luciferase activity of WT-E2F1 induced by miR-4478 overexpression (Figure 6F).

### LLDT-8/WAKMAR2/miR-4478/E2F1/p53 Axis in RA FLS

**Figure 7:**
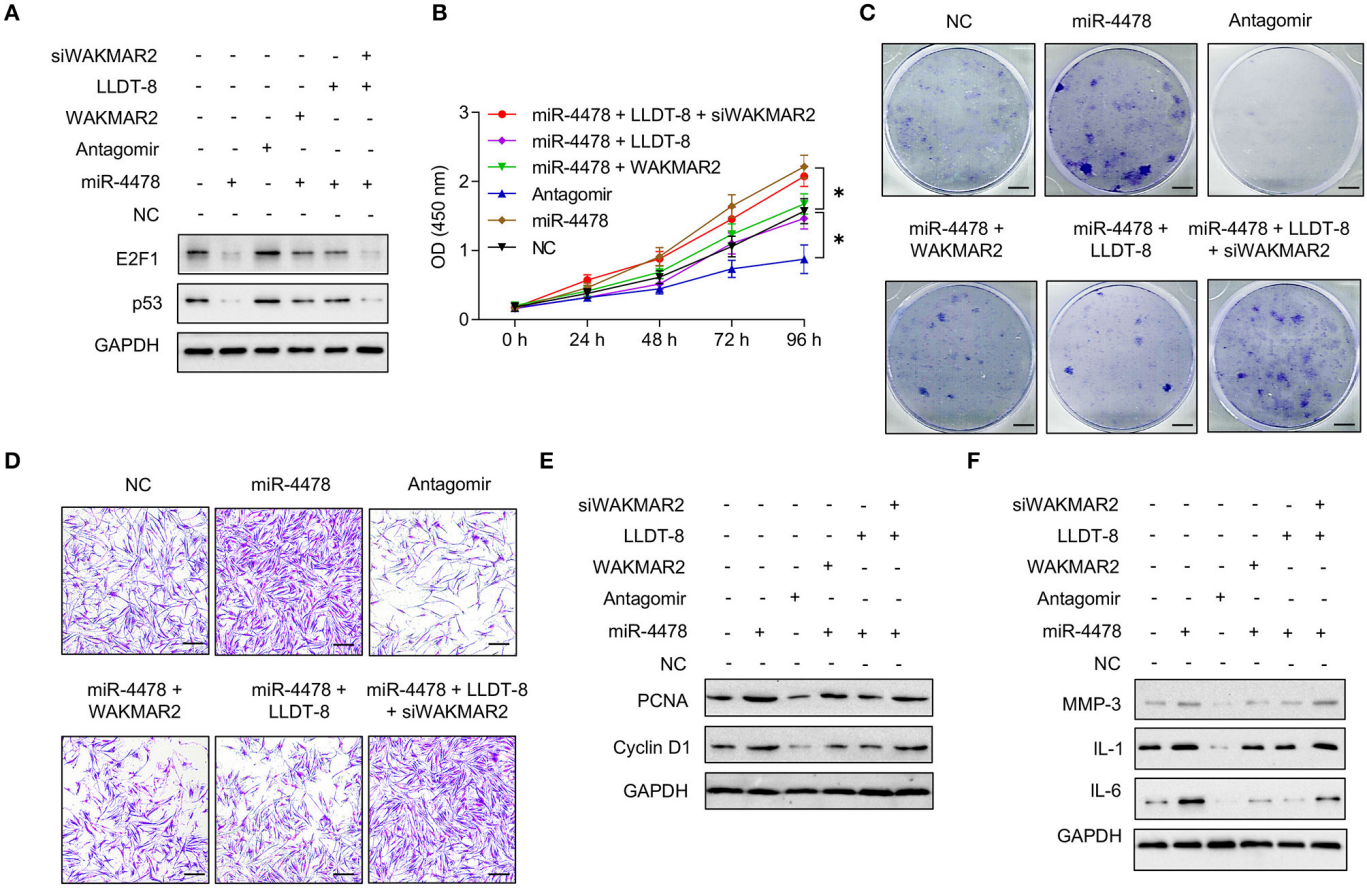
Effects of miR-4478 on RA FLS. **(A)** The levels of p53 and E2F1 in RA FLS transfected with miR-4478 mimic, antagomir, miR-4478 mimic + WAKMAR2 overexpression vector, miR-4478 mimic + LLDT-8 treatment, or miR-4478 mimic +WAKMAR2 silencing vector (siWAKMAR2) + LLDT-8 treatment. **(B)** Proliferative ability of RA FLS with the above treatment, as determined by CCK-8 assay. **(C)** Colony formationof RA FLS with the above treatment. Scale bars = 5 mm. **(D)** Invasion of RA FLS with the above treatment, as determined by transwell assay. Scale bars = 200 μm. **(E)** The levels of PCNA and Cyclin D1 in RA FLS with the above treatment, as determined by western blotting. **(F)** The levels of MMP-3, IL-1, and IL-6 in RA FLS with the above treatment, as determined by western blotting. The data are presented as mean ± SD. *P < 0.05. All experiments were repeated 3 times. RA, rheumatoid arthritis; FLS, fibroblast-like synoviocytes; LLDT-8, (5R)-5-hydroxytriptolide; IL, interleukin; MMP, matrix metalloproteinase; PCNA, proliferating cell nuclear antigen.

**Figure 8:**
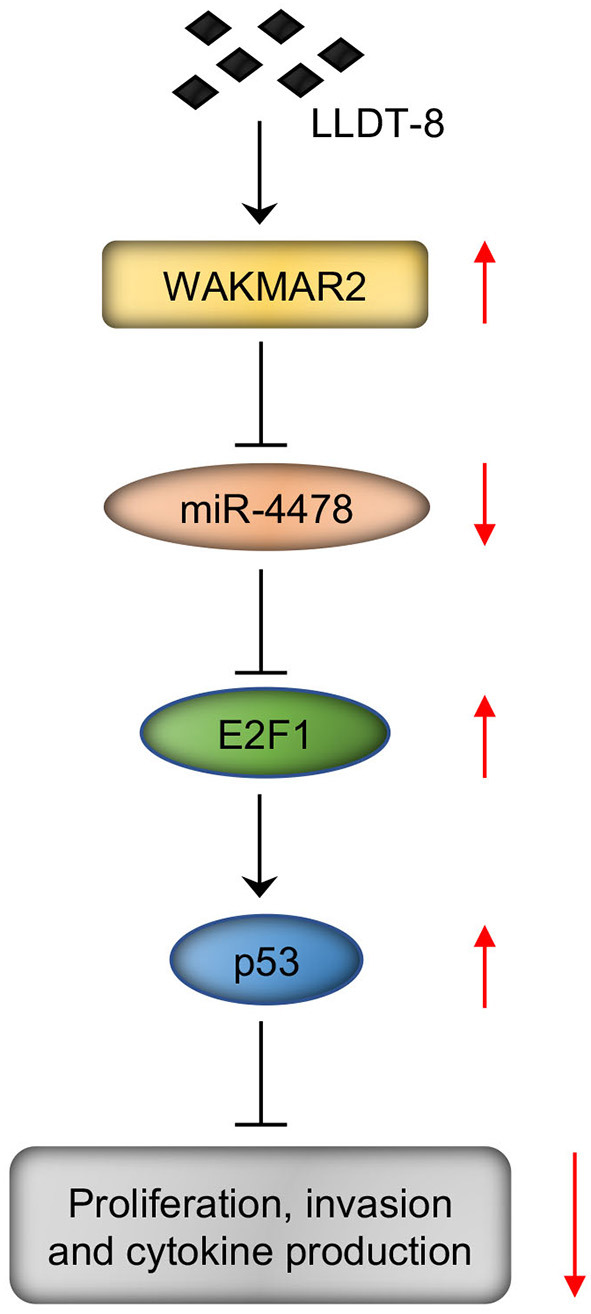
LLDT-8/WAKMAR2/miR-4478/E2F1/p53 axis in RA FLS. Briefly, LLDT-8 induced upregulation of WAKMAR2, which sponged miR-4478, leading to increased expression of E2F1 and p53 and inhibited proliferation and invasion of RA FLS as well as production of cytokines. LLDT-8, (5R)-5-hydroxytriptolide; RA, rheumatoid arthritis; FLS, fibroblast-like synoviocytes.

We examined the role of miR-4478 in RA FLS with or without WAKMAR2 silencing or overexpression or/and LLDT-8 treatment. Overexpression of miR-4478 decreased the levels of E2F1 and p53, whereas silencing of miR-4478 promoted the expression of E2F1 and p53 (Figure 7A and Supplementary Figure 4A). Both enforced expression of WAKMAR2 and LLDT-8 treatment compromised the inhibitory effects of miR-4478 overexpression on levels of E2F1 and p53 (Figure 7A and Supplementary Figure 4A). WAKMAR2 silencing in the presence of LLDT-8 released the inhibitory effects of miR-4478 overexpression on the levels of E2F1 and p53 (Figure 7A and Supplementary Figure 4A). We also examined the proliferation and invasion of RA FLS, expression of cell cycle proteins (PCNA and Cyclin D1), as well as levels of cytokines (MMP-3, IL-1, and IL-6) in RA FLS with the above treatment. Overexpression of miR-4478 promoted proliferation and invasion of RA FLS (Figures 7B–D, Supplementary Figures 4B,C), enhanced the expression of PCNA and Cyclin D1 (Figure 7E and Supplementary Figure 4D), and increased the levels of MMP-3, IL-1, and IL-6 (Figure 7F and Supplementary Figure 4E). Silencing of miR-4478 resulted in adverse effects when compared to miR-4478 overexpression (Figures 7B–F, Supplementary Figures 4B–E). Both enforced expression of WAKMAR2 and the LLDT-8 treatment abolished beneficial effects of miR-4478 overexpression on proliferation and invasion of RA FLS, expression of PCNA and Cyclin D1, as well as the levels of MMP-3, IL-1, and IL-6 (Figures 7B–F, Supplementary Figures 4B,E). WAKMAR2 silencing with the presence of LLDT-8 recovered the beneficial effects of miR-4478 overexpression on proliferation and invasion of RA FLS, expression of PCNA and Cyclin D1, as well as the levels of MMP-3, IL-1, and IL-6 (Figures 7B–F, Supplementary Figures 4B,E).These results demonstrated the essential role of LLDT-8/WAKMAR2/miR-4478/E2F1/p53 axis in RA FLS (Figure 8).

## Discussion

LLDT-8 has a variety of immunosuppressive activities and significant therapeutic effects *in vitro* and *in vivo*. The LLDT-8 prevents collagen-induced arthritis *via* inhibiting OPG/RANK/RANKL signaling in osteoclastogenesis and IFN-gamma signaling in T cells (12, 13). However, it is still unknown whether LLDT-8 regulates FLS function during RA development. In our study, we revealed that LLDT-8 exerted therapeutic effects by inhibiting proliferation and invasion of RA FLS, as well as production of cytokines (MMP-3, IL-1, and IL-6).

Increasing evidence suggests that natural products and small molecules could display therapeutic effects *via* regulating lncRNAs (45). For example, methotrexate decreases NF-κB activity by increasing lincRNA-p21 (46). Tanshinone IIA promotes apoptosis of RA FLS by upregulating GAS5 (47). Quercetin promotes apoptosis of RA FLS through enhancing MALAT1 expression (48). Our study revealed that LLDT-8 upregulated lncRNA WAKMAR2 expression in RA FLS. Gene silencing and overexpression studies demonstrated that WAKMAR2 was a negative regulator of tumor-like phenotypes of RA FLS. Silencing of WAKMAR2 abolished the therapeutic effects of LLDT-8 on RA FLS, implying that the therapeutic effects of LLDT-8 might be dependent on WAKMAR2.

In our study, we established a link between LLDT-8 and WAKMAR2. To date, no work reports the direct target of LLDT-8. The underlying mechanism behind upregulation of WAKMAR2 expression by LLDT-8 is still unknown. But in general, LLDT-8 would directly target WAKMAR2 or indirectly affect WAKMAR2 expression *via* targeting other molecules. This needs to be addressed in future studies. Efforts toward understanding fundamental principles of small molecule drug-RNA recognition combined with advances in methodology development should pave the way toward targeting lncRNAs (49). Currently, structure-based methods such as molecular docking could not be used for predicting the exact structure of lncRNAs and interpreting direct interaction between small molecule drugs and lncRNAs. But the recently developed structural biology, mass spectroscopy, and pattern recognition have made it possible to detect small molecule drugs that directly bind to lncRNA (50, 51). If small molecule drugs affect lncRNA expression *via* targeting other molecules rather than directly targeting lncRNAs, the construction of a small molecule drug-lncRNA network could be helpful to find the molecules that mediate the indirect interaction between small molecule drugs and lncRNAs (45).

Identification of aberrantly expressed lncRNAs in RA and exploration of the underlying molecular mechanisms will offer a new direction to understand the pathogenesis of RA (52). To date, LncRNA H19, Hotair, lincRNA-p21, C5T1, LOC100652951, and LOC100506036 have been verified to be dysregulated in T cells, PBMCs, exosomes, and synovial cells, which are associated with inflammation and immune reaction in RA (52). The WAKMAR2 has been proven as an important regulator in the healing of skin wound and its deficiency may contribute to the pathogenesis of chronic wounds (53). In future, it is necessary to examine the pathological role of WAKMAR2 in RA development and to propose whether WAKMAR2 could be a promising therapeutic target for RA treatment.

To determine the regulatory network of LLDT-8/WAKMAR2, we performed miRNA microarray in RA FLS treated with LLDT-8 and conducted bioinformatics analysis. We predicted and validated that p53 signaling was the downstream pathway of LLDT-8/WAKMAR2. To explore the mechanism of LLDT-8/WAKMAR2 in the regulation of p53 pathway, we analyzed the target miRNAs of WAKMAR2. Our data showed that LLDT-8 and WAKMAR2 inhibited miR-4478 expression in RA FLS. The luciferase reporter assay suggested that WAKMAR2 sponged miR-4478 and prevented the interaction between miR-4478 and target gene E2F1. The p53 is a tumor suppressor that limits cell proliferation and survival through regulating cell cycle (54). The E2F1 can activate p53 transcription (43, 44). We found that miR-4478 inhibited E2F1 and p53 expression and promoted tumor-like phenotypes of RA FLS. Both enforced expression of WAKMAR2 and LLDT-8 treatment compromised the inhibitory effects of miR-4478 on E2F1 and p53 expression and abolished the beneficial effects of miR-4478 on RA FLS. WAKMAR2 silencing in the presence of LLDT-8 released the inhibitory effects of miR-4478 on E2F1 and p53expression and recovered the beneficial effects of miR-4478 on RA FLS, suggesting that WAKMAR2/miR-4478/E2F1/p53 axis is essential for the action of LLDT-8 on RA FLS.

In conclusion, LLDT-8 inhibited proliferation and invasion of RA FLS, as well as the production of cytokines *via* WAKMAR2/miR-4478/E2F1/p53 axis.

## Data Availability Statement

The raw data supporting the conclusions of this article will be made available by the authors, without undue reservation.

## Ethics Statement

The studies involving human participants were reviewed and approved by the Ethical Committee of Guanghua Hospital. The patients/participants provided their written informed consent to participate in this study.

## Author Contributions

CL and DH jointly supervised the whole project. XZ and DX performed the major research and wrote the paper in equal contribution. JH, AL, RW, YJ, RZ, CC, LingX, LinsX, and JF provided the technical support and professional expertise. All authors contributed to the article and approved the submitted version.

## Conflict of Interest

The authors declare that the research was conducted in the absence of any commercial or financial relationships that could be construed as a potential conflict of interest.
