# Supplementary material for: Therapeutic Effects of (5R)-5-Hydroxytriptolide on Fibroblast-Like Synoviocytes in Rheumatoid Arthritis via lncRNA WAKMAR2/miR-4478/E2F1/p53 Axis
Source: Front Immunol. 2021 Feb 16;12:605616. doi: 10.3389/fimmu.2021.605616 (PMC7921149; doi:10.3389/fimmu.2021.605616)
Supplement: Supplementary file 1 [file Data_Sheet_1.pdf]

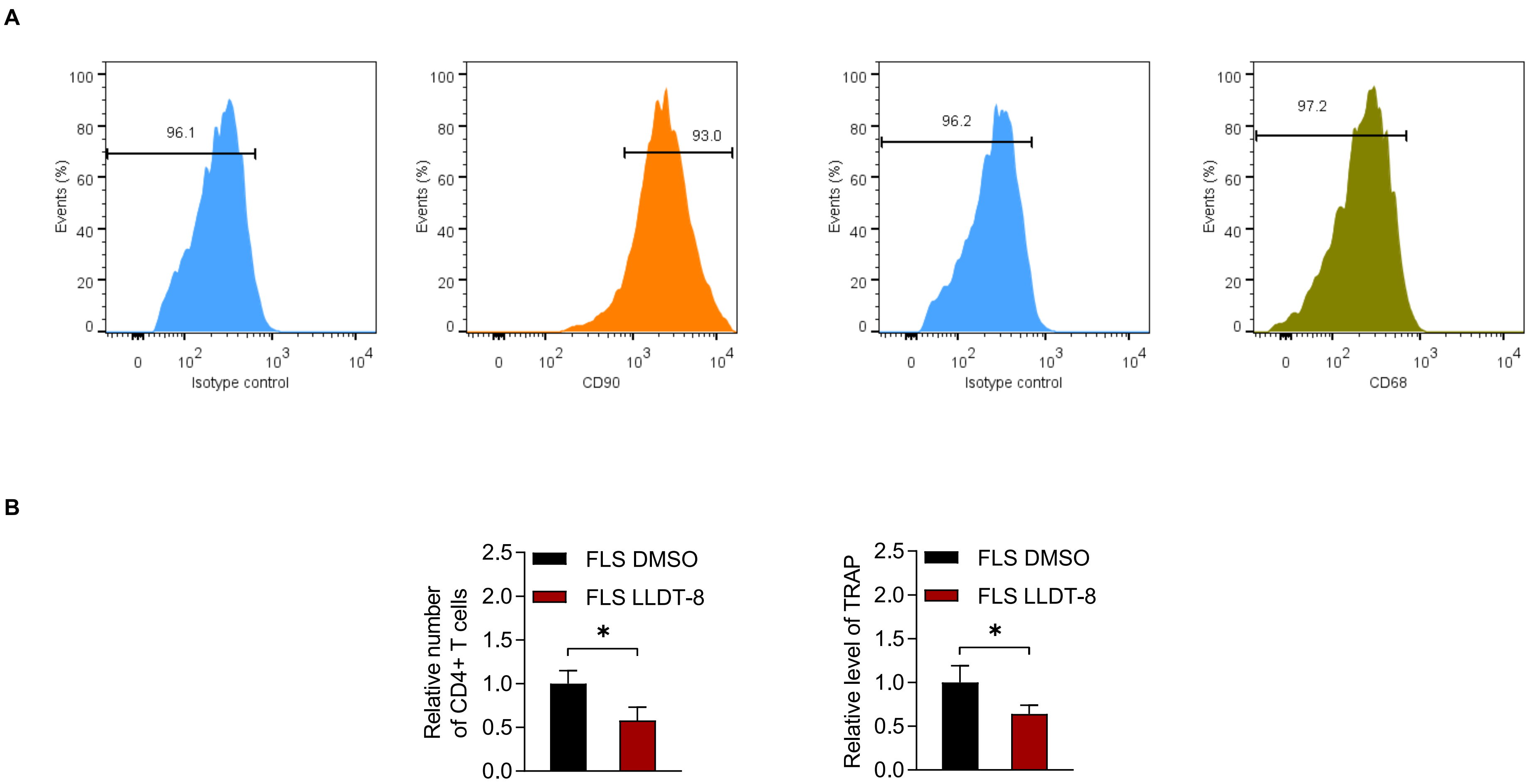
A
B
Events (%)
96.1
Isotype control
93.0
CD90
96.2
Isotype control
97.2
CD68
FLS DMSO
FLS LLDT-8
Relative number of CD4+ T cells
Relative level of TRAP
*

Supplementary Fig. 1 Characterization of RA FLS and cell co-culture. (A) Expression of cell surface markers (CD90 and CD68) and isotype controls on RA FLS, as determined by flow cytometry. **(B)** Co-culture of $CD4^+$ T cells or $CD14^+$ monocytes/macrophages with RA FLS. Before cell co-culture, RA FLS were treated with LLDT-8 or DMSO. Proliferative ability of $CD4^+$ T cells were examined by flow cytometry. TRAP expression in $CD14^+$ monocytes/macrophages were determined by ELISA. Data are presented as mean ± SD. $^*P < 0.05$. All experiments were repeated three times.

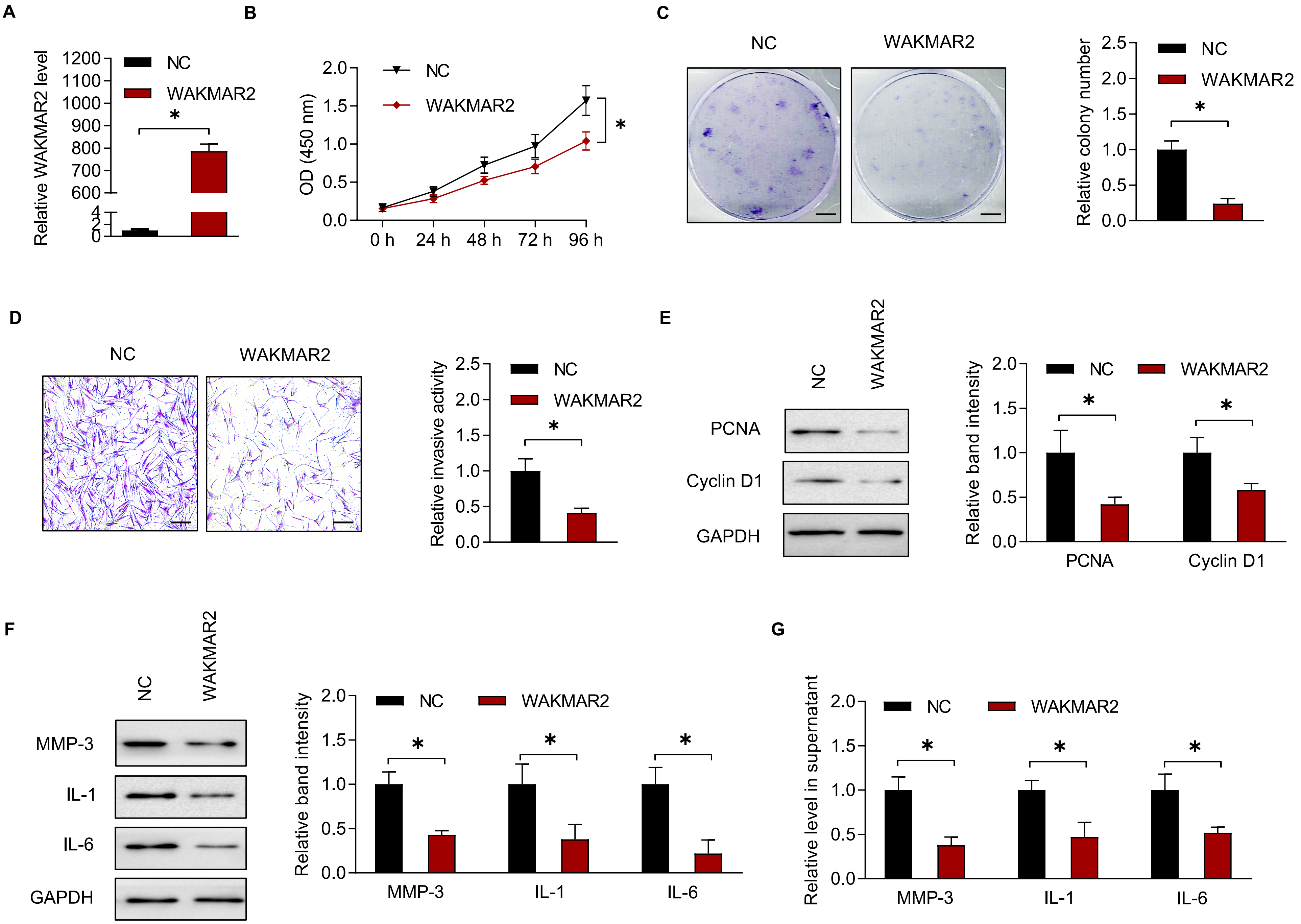

A
Relative WAKMAR2 level
NC
WAKMAR2
*
B
OD (450 nm)
NC
WAKMAR2
0 h
24 h
48 h
72 h
96 h
*
C
NC
WAKMAR2
Relative colony number
*
D
NC
WAKMAR2
Relative invasive activity
*
E
NC
WAKMAR2
PCNA
Cyclin D1
GAPDH
Relative band intensity
*
F
NC
WAKMAR2
MMP-3
IL-1
IL-6
GAPDH
Relative band intensity
*
G
Relative level in supernatant
NC
WAKMAR2
MMP-3
IL-1
IL-6
*

Supplementary Fig. 2 Effects of WAKMAR2 overexpression on RA FLS. (A) Level of WAKMAR2 in RA FLS transfected with WAKMAR2 overexpressing vector or negative control vector (NC), as determined by real-time PCR. **(B)** Proliferative ability of RA FLS with the above transfection, as determined by CCK-8 assay. **(C)** Colony formation of RA FLS with the above transfection. **(D)** Invasion of RA FLS with the above transfection, as determined by transwell assay. Scale bars = 200 μm. **(E)** The levels of PCNA and Cyclin D1 in RA FLS with the above transfection, as determined by western blotting. **(F)** The levels of MMP-3, IL-1 and IL-6 in RA FLS with the above transfection, as determined by western blotting. **(G)** The levels of MMP-3, IL-1 and IL-6 in supernatant of RA FLS with the above transfection, as determined by ELISA. Data are presented as mean ± SD. $^*P < 0.05$. Both representative images and quantitative measurement of colony formation, invasion and western blotting were shown. All experiments were repeated three times.

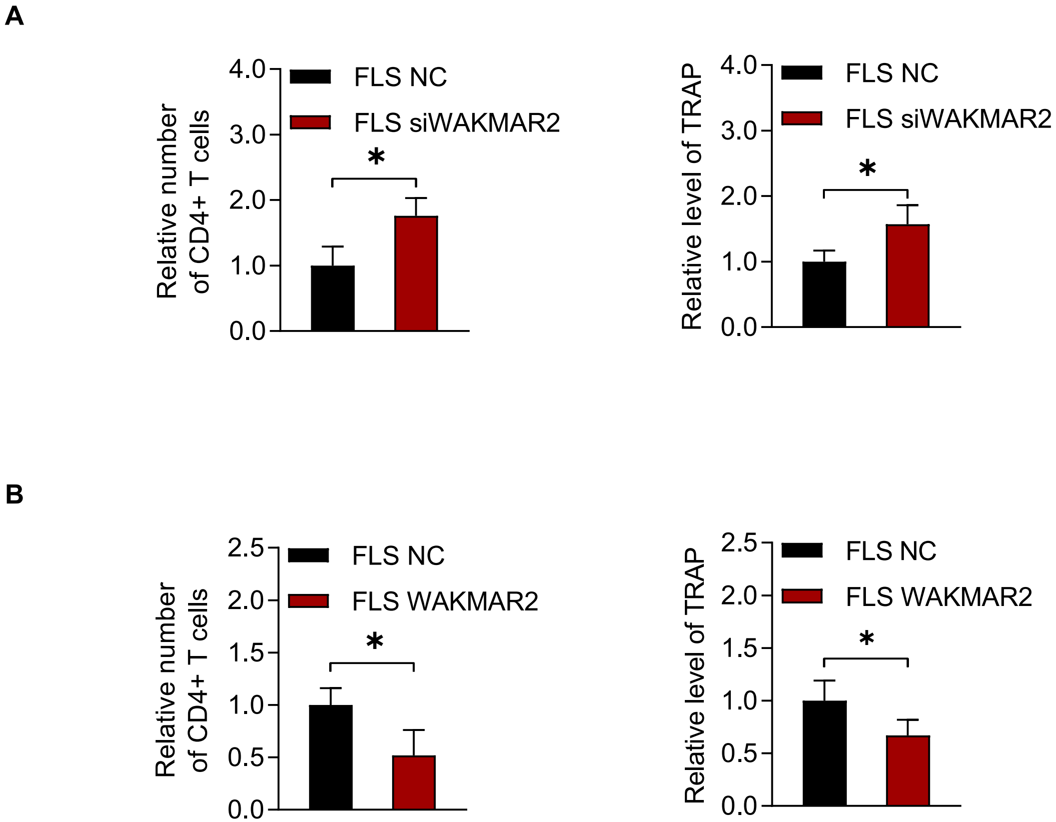
A
4.0
3.0
2.0
1.0
0.0
Relative number of CD4+ T cells
FLS NC
FLS siWAKMAR2
*
Relative level of TRAP
FLS NC
FLS siWAKMAR2
*
B
2.5
2.0
1.5
1.0
0.5
0.0
Relative number of CD4+ T cells
FLS NC
FLS WAKMAR2
*
Relative level of TRAP
FLS NC
FLS WAKMAR2
*

Supplementary Fig. 3 Co-culture of T cells or monocytes/macrophages with RA FLS. (A) Co-culture of $CD4^+$ T cells or $CD14^+$ monocytes/macrophages with RA FLS transfected with WAKMAR2 silencing vector (siWAKMAR2) or negative control vector (NC). **(B)** Co-culture of $CD4^+$ T cells or $CD14^+$ monocytes/macrophages with RA FLS transfected with WAKMAR2 overexpressing vector or negative control vector (NC). Proliferative ability of $CD4^+$ T cells were examined by flow cytometry. TRAP expression in $CD14^+$ monocytes/macrophages were determined by ELISA. Data are presented as mean ± SD. *$P < 0.05$. All experiments were repeated three times.

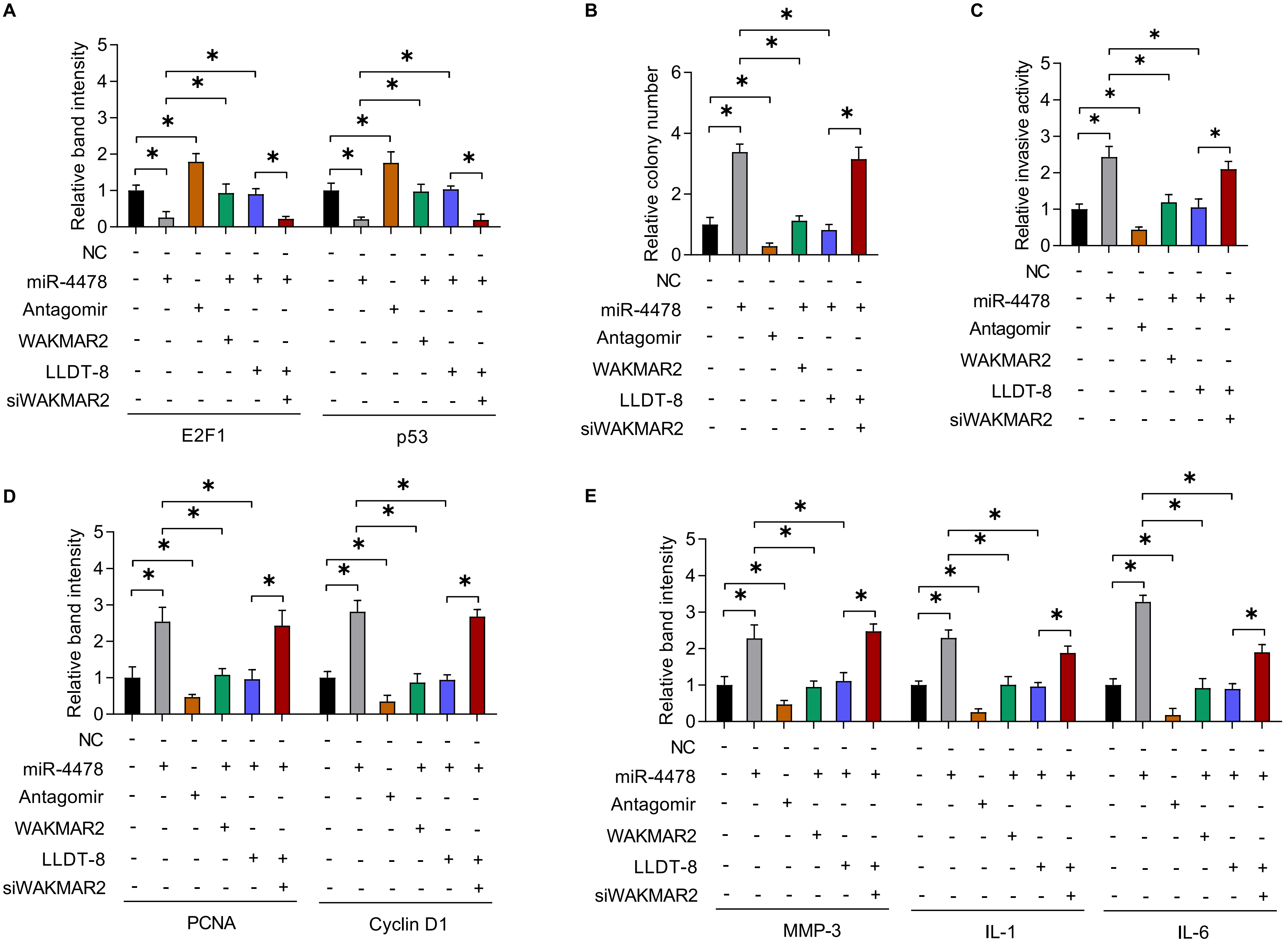

A
Relative band intensity
NC
miR-4478
Antagomir
WAKMAR2
LLDT-8
siWAKMAR2
E2F1
p53
B
Relative colony number
C
Relative invasive activity
D
PCNA
Cyclin D1
E
MMP-3
IL-1
IL-6

Supplementary Fig. 4 Quantitative measurement in RA FLS. (A) Levels of p53 and E2F1 in RA FLS transfected with miR-4478 mimic, antagomir, miR-4478 mimic + WAKMAR2 overexpression vector, miR-4478 mimic + LLDT-8 treatment or miR-4478 mimic + WAKMAR2 silencing vector (siWAKMAR2) + LLDT-8 treatment. **(B)** Colony formation of RA FLS with the above treatment. **(C)** Invasion of RA FLS with the above treatment. **(D)** Levels of PCNA and Cyclin D1 in RA FLS with the above treatment. **(E)** Levels of MMP-3, IL-1 and IL-6 in RA FLS with the above treatment. Data are presented as mean ± SD. $*P < 0.05$. All experiments were repeated three times.
